# Supplementary material for: Cardiovascular Disease-Associated MicroRNA Dysregulation during the First Trimester of Gestation in Women with Chronic Hypertension and Normotensive Women Subsequently Developing Gestational Hypertension or Preeclampsia with or without Fetal Growth Restriction
Source: Biomedicines. 2022 Jan 25;10(2):256. doi: 10.3390/biomedicines10020256 (PMC8869238; doi:10.3390/biomedicines10020256)
Supplement: Supplementary file 1 [file biomedicines-10-00256-s001.zip › biomedicines-1539289-supplementary/Supplementary Figure S1.pdf]

Supplementary Figure S1.

A

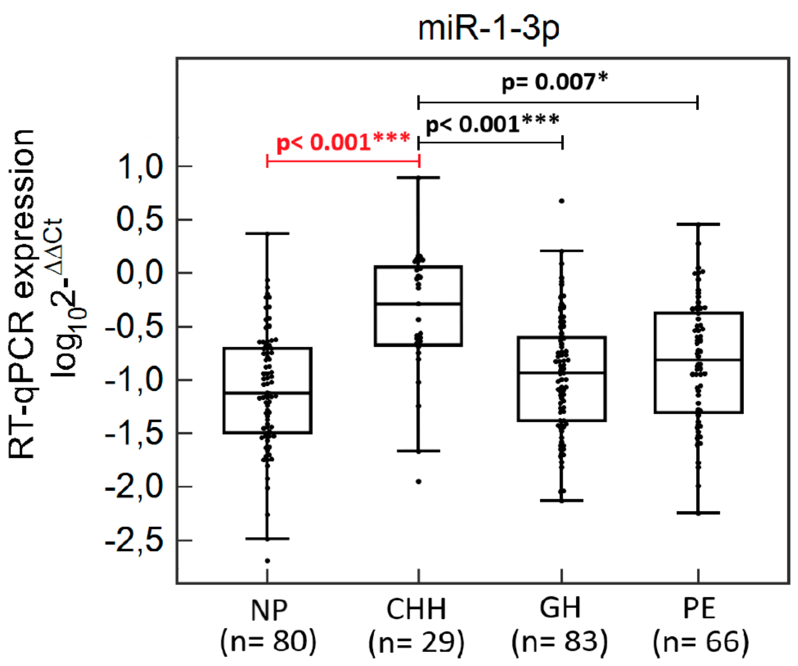

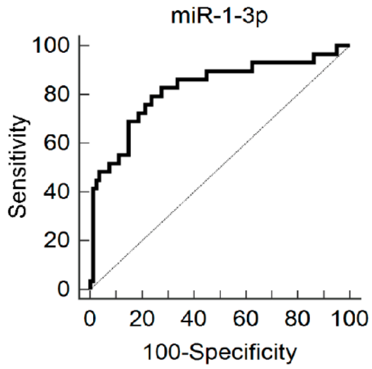

Area under the ROC curve (AUC)	0.823
Standard Error	0.0507
95% Confidence interval	0.739 to 0.890
Significance level P (Area=0.5)	<0.0001

Estimated sensitivity at fixed specificity			
Specificity	Sensitivity	95% CI	Criterion
90.00	51.72	29.63 to 72.41	>0.377841

Criterion	Sensitivity	95% CI	Specificity	95% CI	+LR	95% CI	-LR	95% CI
>0.199151	79.31	60.3 - 92.0	76.25	65.4 - 85.1	3.34	2.2 - 5.2	0.27	0.1 - 0.6

B

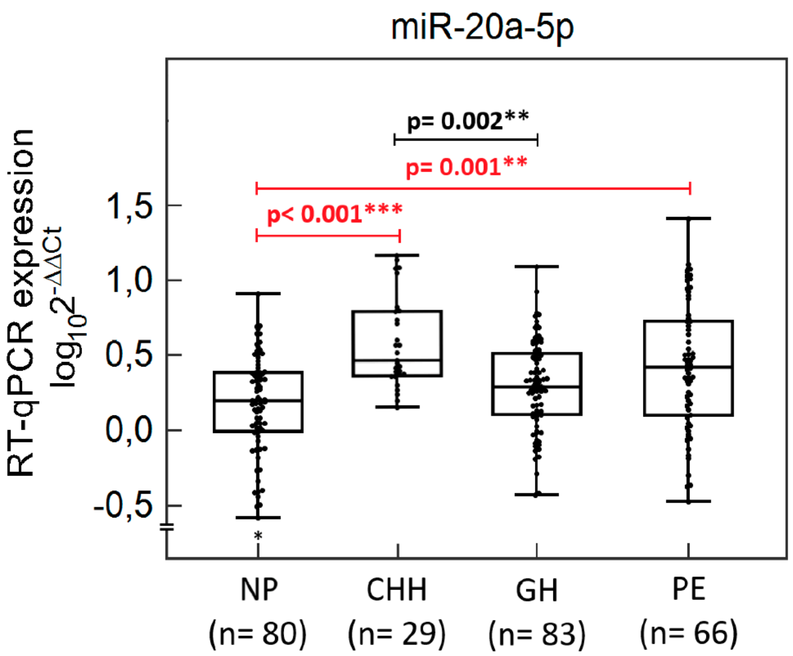

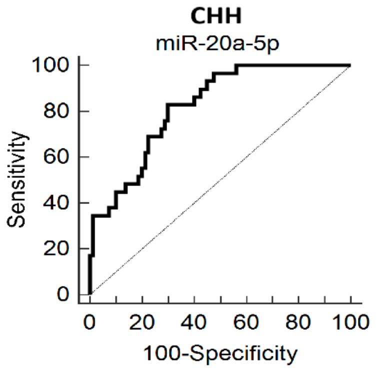

Area under the ROC curve (AUC)	0.820
Standard Error	0.0410
95% Confidence interval	0.735 to 0.887
Significance level P (Area=0.5)	<0.0001

Estimated sensitivity at fixed specificity			
Specificity	Sensitivity	95% CI	Criterion
90.00	44.83	27.59 to 66.90	>3.587078

Criterion	Sensitivity	95% CI	Specificity	95% CI	+LR	95% CI	-LR	95% CI
>2.302443	82.76	64.2 - 94.2	70.00	58.7 - 79.7	2.76	1.9 - 4.0	0.25	0.1 - 0.6

C

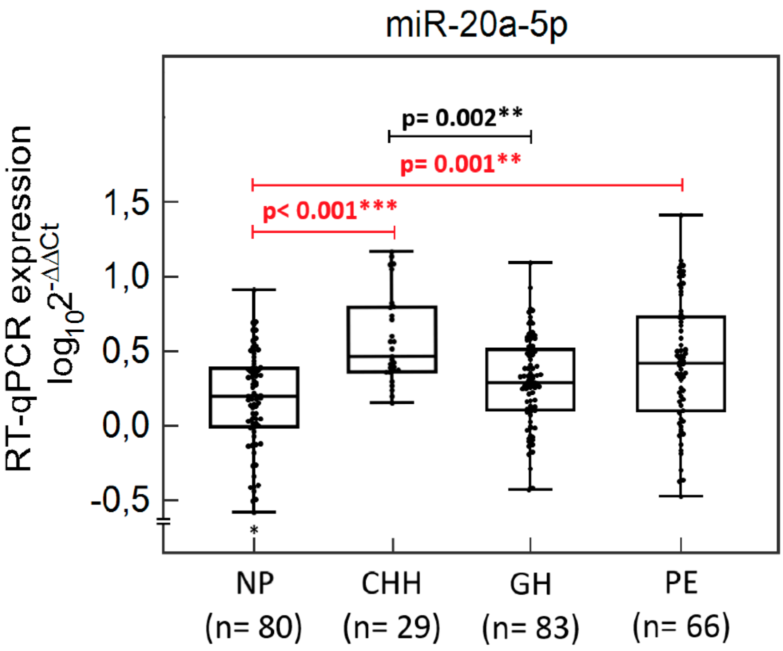

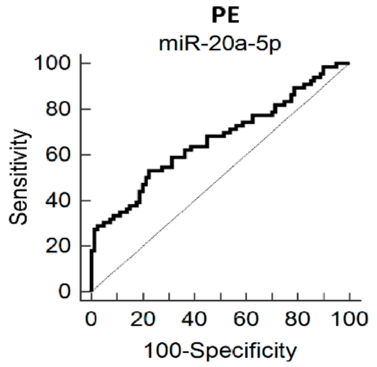

Area under the ROC curve (AUC)	0.666
Standard Error	0.0459
95% Confidence interval	0.584 to 0.742
Significance level P (Area=0.5)	0.0003

Estimated sensitivity at fixed specificity			
Specificity	Sensitivity	95% CI	Criterion
90.00	33.33	22.73 to 46.97	>3.587078

Criterion	Sensitivity	95% CI	Specificity	95% CI	+LR	95% CI	-LR	95% CI
>2.43637	53.03	40.3 - 65.4	77.50	66.8 - 86.1	2.36	1.5 - 3.8	0.61	0.5 - 0.8

D

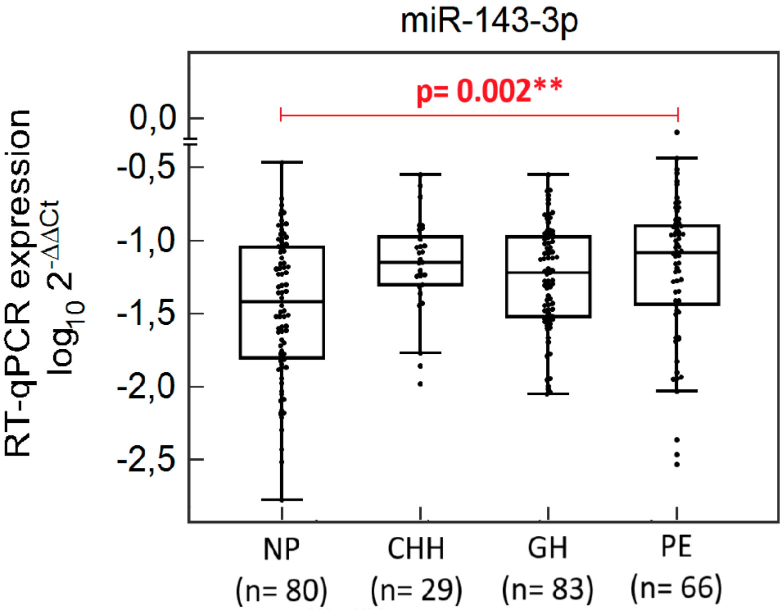

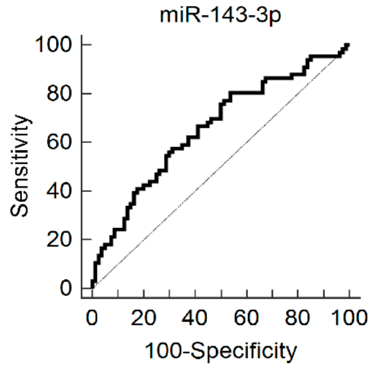

Area under the ROC curve (AUC)	0.659
Standard Error	0.0456
95% Confidence interval	0.576 to 0.736
z statistic	3.493
Significance level P (Area=0.5)	0.0005

Estimated sensitivity at fixed specificity			
Specificity	Sensitivity	95% CI	Criterion
90.00	24.24	12.12 to 42.42	>0.129856

Criterion	Sensitivity	95% CI	Specificity	95% CI	+LR	95% CI	-LR	95% CI
>0.031066	80.30	68.7 - 89.1	46.25	35.0 - 57.8	1.49	1.2 - 1.9	0.43	0.2 - 0.7

E

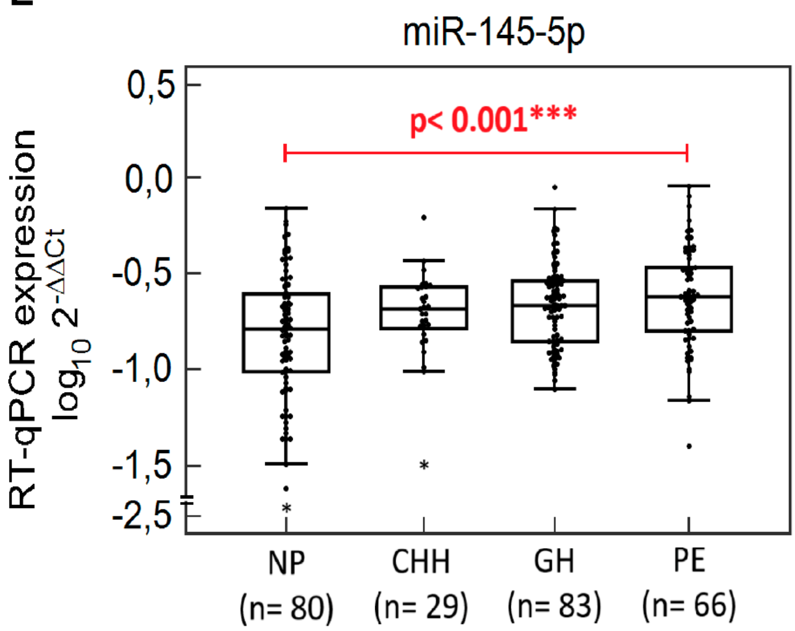

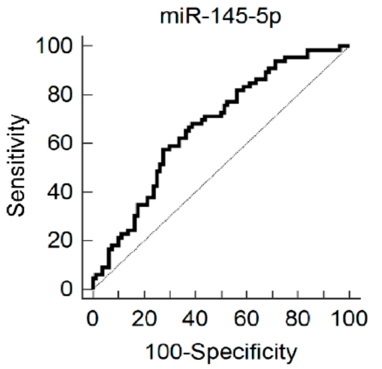

Area under the ROC curve (AUC)	0.673
Standard Error	0.0444
95% Confidence interval	0.590 to 0.748
Significance level P (Area=0.5)	0.0001

Estimated sensitivity at fixed specificity			
Specificity	Sensitivity	95% CI	Criterion
90.00	21.21	9.09 to 37.88	>0.403208

Criterion	Sensitivity	95% CI	Specificity	95% CI	+LR	95% CI	-LR	95% CI
>0.223013	57.58	44.8 - 69.7	72.50	61.4 - 81.9	2.09	1.4 - 3.2	0.59	0.4 - 0.8

F

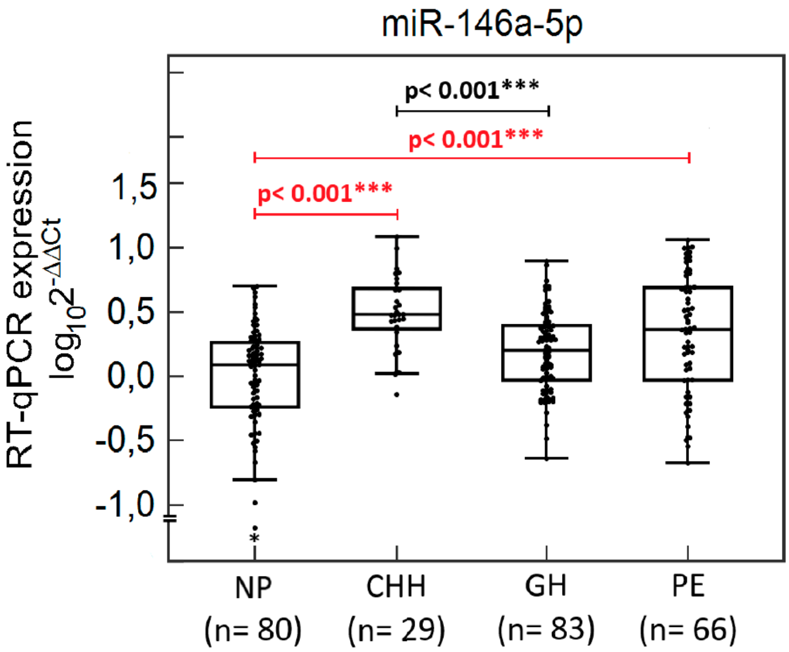

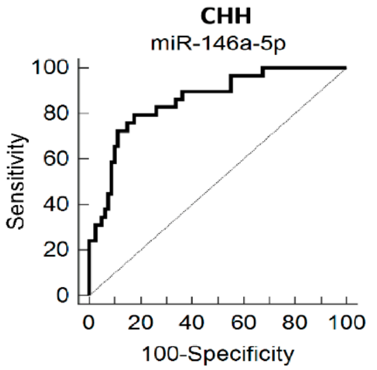

Area under the ROC curve (AUC)	0.857
Standard Error	0.0400
95% Confidence interval	0.777 to 0.917
Significance level P (Area=0.5)	<0.0001

Estimated sensitivity at fixed specificity			
Specificity	Sensitivity	95% CI	Criterion
90.00	65.52	27.59 to 89.66	>2.744534

Criterion	Sensitivity	95% CI	Specificity	95% CI	+LR	95% CI	-LR	95% CI
>2.073306	79.31	60.3 - 92.0	82.50	72.4 - 90.1	4.53	2.7 - 7.6	0.25	0.1 - 0.5

G

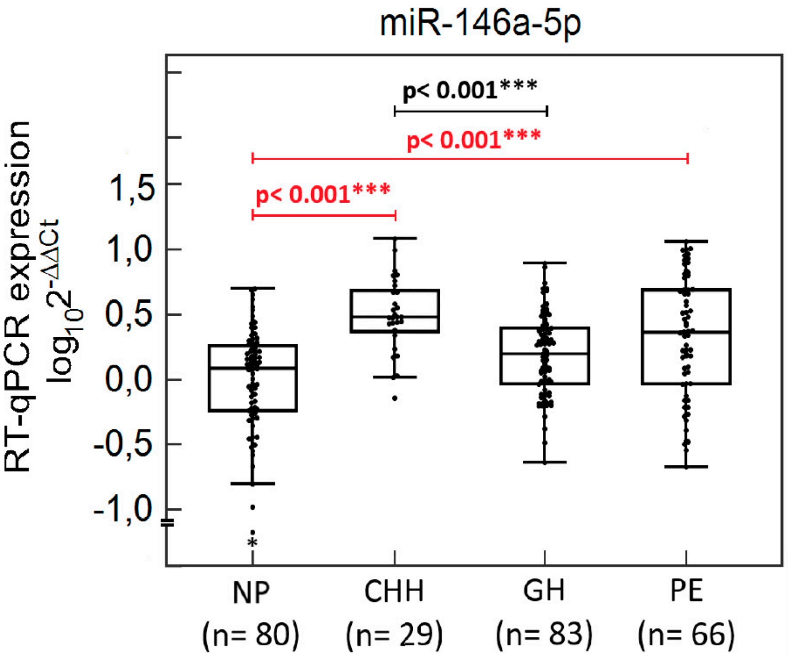

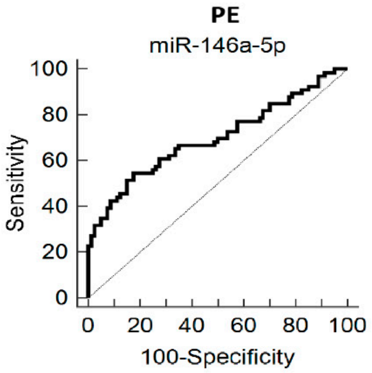

Area under the ROC curve (AUC)	0.698
Standard Error	0.0450
95% Confidence interval	0.617 to 0.771
z statistic	4.399
Significance level P (Area=0.5)	<0.0001

Estimated sensitivity at fixed specificity			
Specificity	Sensitivity	95% CI	Criterion
90.00	42.42	27.27 to 57.58	>2.744534

Criterion	Sensitivity	95% CI	Specificity	95% CI	+LR	95% CI	-LR	95% CI
>2.073306	54.55	41.8 - 66.9	82.50	72.4 - 90.1	3.12	1.8 - 5.3	0.55	0.4 - 0.7

H

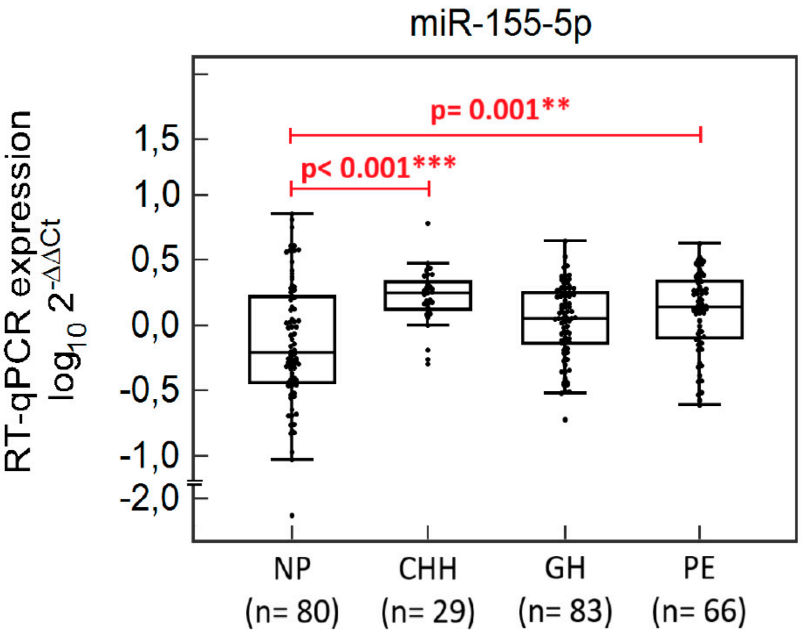

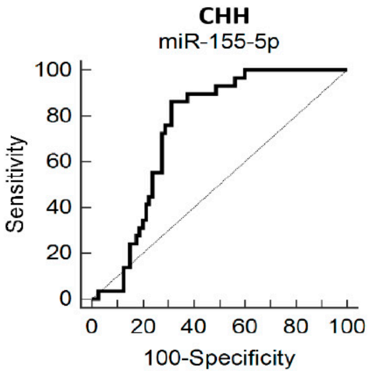

Area under the ROC curve (AUC)	0.745
Standard Error	0.0462
95% Confidence interval	0.652 to 0.824
Significance level P (Area=0.5)	<0.0001

Estimated sensitivity at fixed specificity			
Specificity	Sensitivity	95% CI	Criterion
90.00	3.45	0.00 to 34.48	>3.615596

Criterion	Sensitivity	95% CI	Specificity	95% CI	+LR	95% CI	-LR	95% CI
>1.103463	86.21	68.3 - 96.1	68.75	57.4 - 78.7	2.76	1.9 - 3.9	0.20	0.08 - 0.5

I

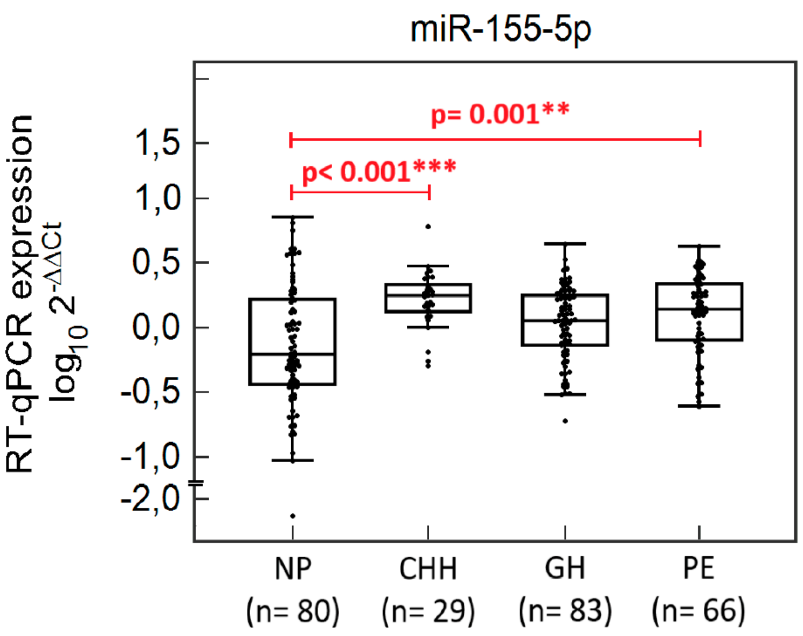

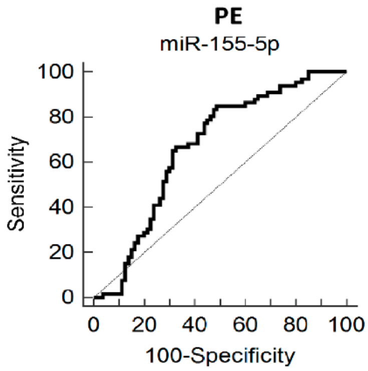

Area under the ROC curve (AUC)	0.667
Standard Erro	0.0455
95% Confidence interval	0.584 to 0.742
Significance level P (Area=0.5)	0.0002

Estimated sensitivity at fixed specificity			
Specificity	Sensitivity	95% CI	Criterion
90.00	1.52	0.00 to 24.24	>3.615596

Criterion	Sensitivity	95% CI	Specificity	95% CI	+LR	95% CI	-LR	95% CI
>0.617536	84.85	73.9 - 92.5	51.25	39.8 - 62.6	1.74	1.4 - 2.2	0.30	0.2 - 0.5

J

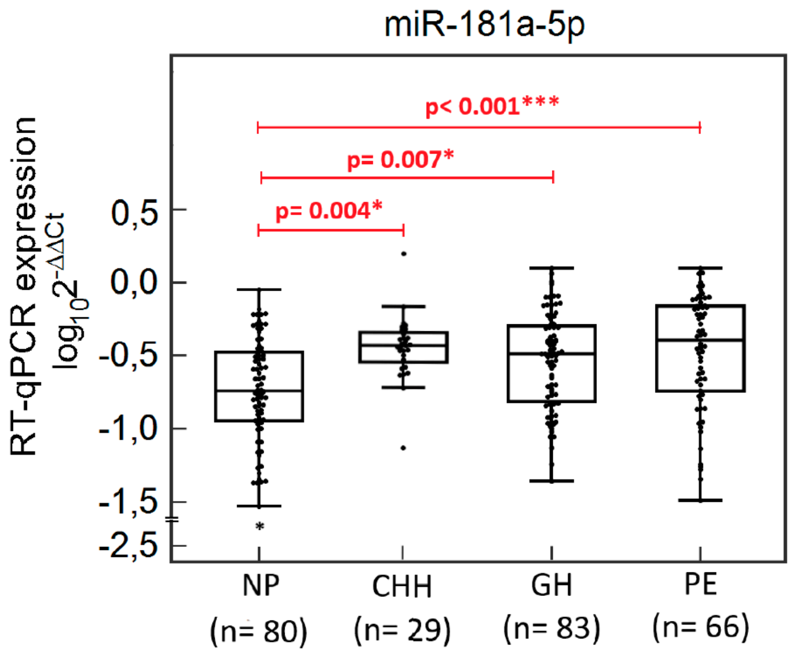

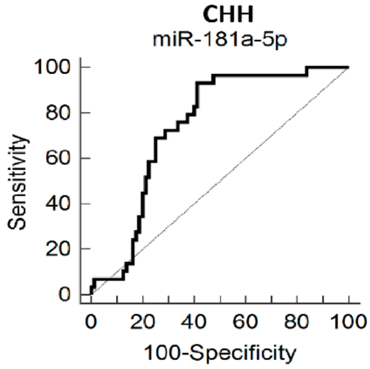

Area under the ROC curve (AUC)	0.742
Standard Error	0.0481
95% Confidence interval	0.649 to 0.821
Significance level P (Area=0.5)	<0.0001

Estimated sensitivity at fixed specificity			
Specificity	Sensitivity	95% CI	Criterion
90.00	6.90	0.00 to 31.03	>0.521629

Criterion	Sensitivity	95% CI	Specificity	95% CI	+LR	95% CI	-LR	95% CI
>0.222962	93.10	77.2 - 99.2	58.75	47.2 - 69.6	2.26	1.7 - 3.0	0.12	0.03 - 0.5

K

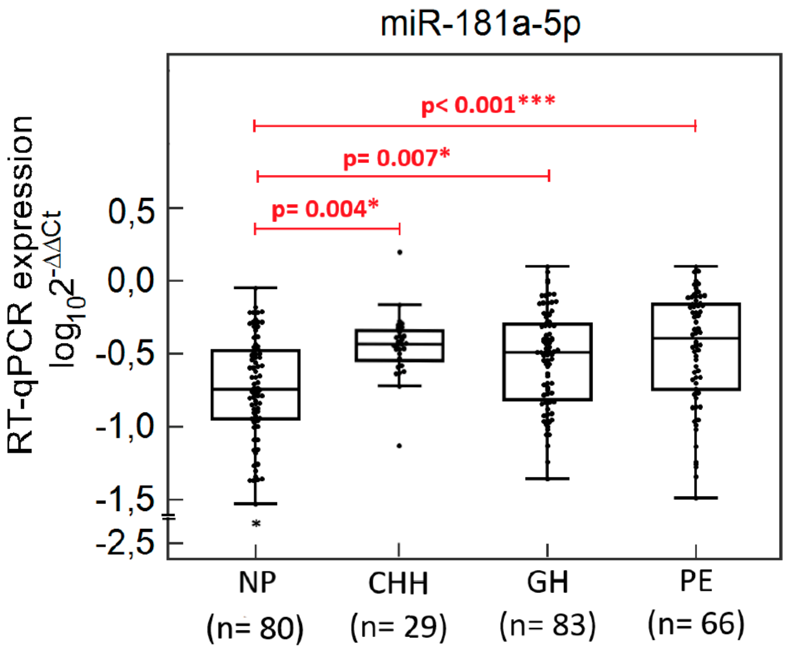

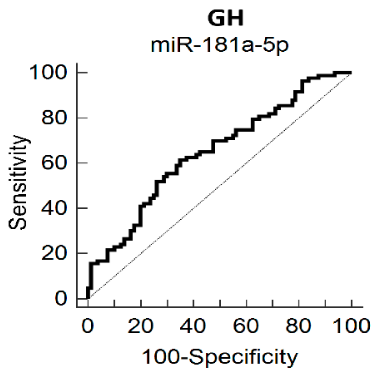

Area under the ROC curve (AUC)	0.649
Standard Error	0.0429
95% Confidence interval	0.570 to 0.722
Significance level P (Area=0.5)	0.0005

Estimated sensitivity at fixed specificity			
Specificity	Sensitivity	95% CI	Criterion
90.00	22.89	12.18 to 36.14	>0.521629

Criterion	Sensitivity	95% CI	Specificity	95% CI	+LR	95% CI	-LR	95% CI
>0.261811	61.45	50.1 - 71.9	65.00	53.5 - 75.3	1.76	1.2 - 2.5	0.59	0.4 - 0.8

L

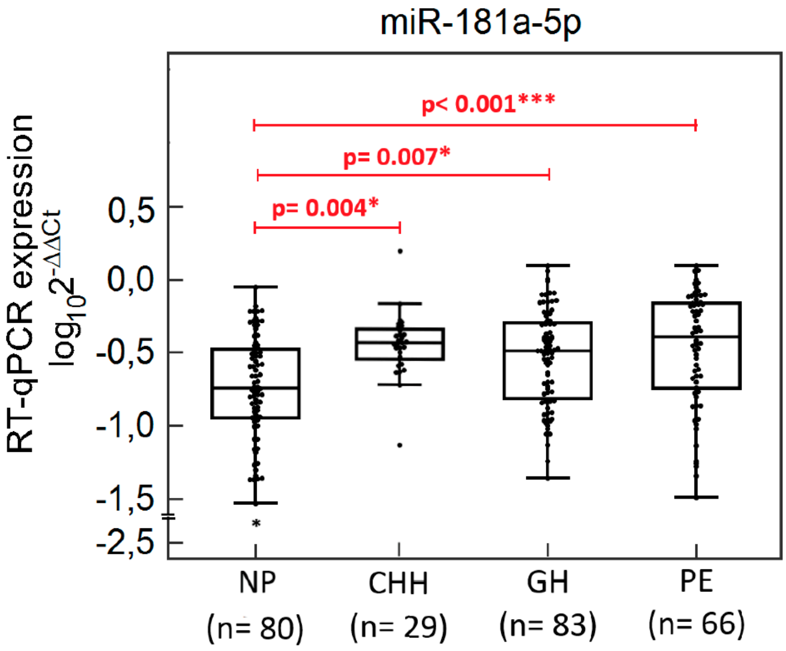

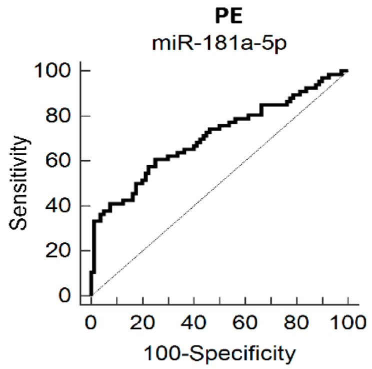

Area under the ROC curve (AUC)	0.707
Standard Error	0.0442
95% Confidence interval	0.626 to 0.779
Significance level P (Area=0.5)	<0.0001

Estimated sensitivity at fixed specificity			
Specificity	Sensitivity	95% CI	Criterion
90.00	40.91	29.54 to 54.55	>0.521629

Criterion	Sensitivity	95% CI	Specificity	95% CI	+LR	95% CI	-LR	95% CI
>0.32734	60.61	47.8 - 72.4	75.00	64.1 - 84.0	2.42	1.6 - 3.7	0.53	0.4 - 0.7

M

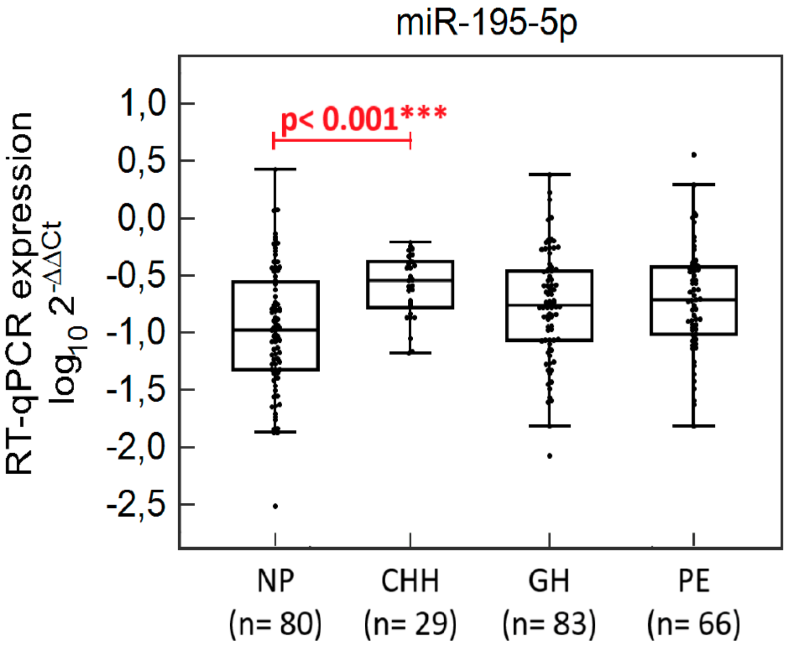

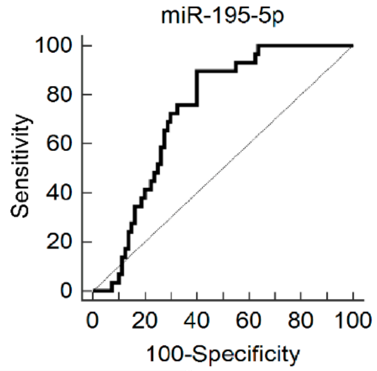

Area under the ROC curve (AUC)	0.732
Standard Error	0.0475
95% Confidence interval	0.639 to 0.812
Significance level P (Area=0.5)	<0.0001

Estimated sensitivity at fixed specificity			
Specificity	Sensitivity	95% CI	Criterion
90.00	6.90	0.00 to 37.93	>0.553901

Criterion	Sensitivity	95% CI	Specificity	95% CI	+LR	95% CI	-LR	95% CI
>0.135	89.66	72.6 - 97.8	60.00	48.4 - 70.8	2.24	1.7 - 3.0	0.17	0.06 - 0.5

N

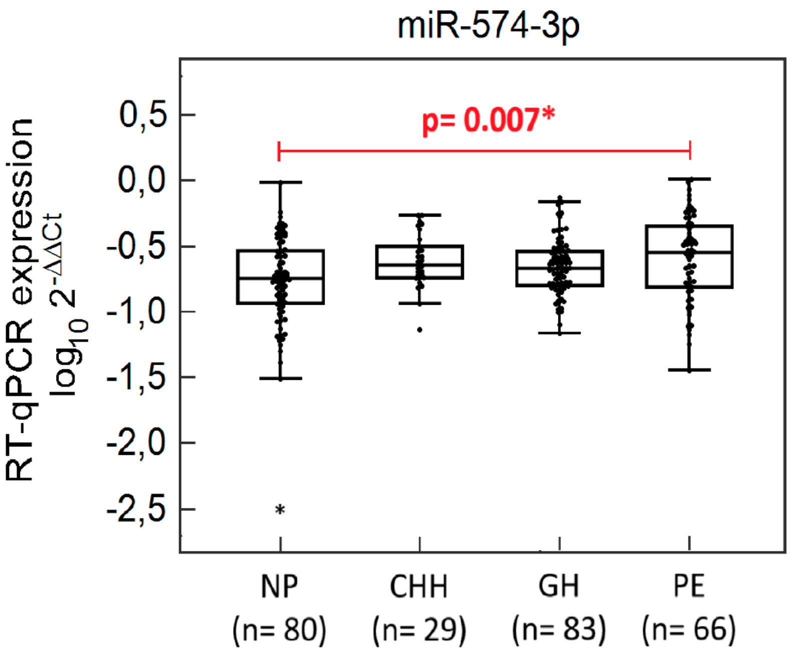

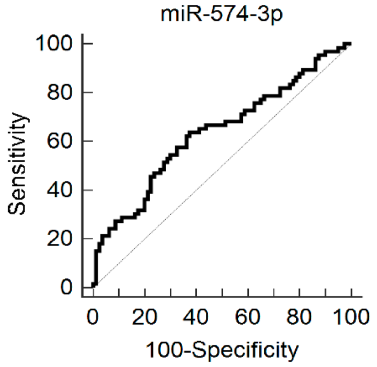

Area under the ROC curve (AUC)	0.636
Standard Error	0.0467
95% Confidence interval	0.553 to 0.714
Significance level P (Area=0.5)	0.0035

Estimated sensitivity at fixed specificity			
Specificity	Sensitivity	95% CI	Criterion
90.00	27.27	18.18 to 42.42	>0.42623

Criterion	Sensitivity	95% CI	Specificity	95% CI	+LR	95% CI	-LR	95% CI
>0.202286	63.64	50.9 - 75.1	62.50	51.0 - 73.1	1.70	1.2 - 2.4	0.58	0.4 - 0.8

Supplementary Figure S1. Gene expression of cardiovascular disease associated microRNAs in peripheral blood leukocytes in early stages of gestation – comparison between NP, CHH, GH and PE – statistical significant data after Benjamini-Hochberg correction (results after the Benjamini-Hochberg correction are marked by * for α = 0.05, ** for α = 0.01, and *** for α = 0.001). NP, normal pregnancies; CHH, chronic hypertension; GH, gestational hypertension; PE, preeclampsia.
