# Supplementary material for: Cardiovascular Disease-Associated MicroRNA Dysregulation during the First Trimester of Gestation in Women with Chronic Hypertension and Normotensive Women Subsequently Developing Gestational Hypertension or Preeclampsia with or without Fetal Growth Restriction
Source: Biomedicines. 2022 Jan 25;10(2):256. doi: 10.3390/biomedicines10020256 (PMC8869238; doi:10.3390/biomedicines10020256)
Supplement: Supplementary file 1 [file biomedicines-10-00256-s001.zip › biomedicines-1539289-supplementary/Supplementary Figure S2.pdf]

Supplementary Figure S2.

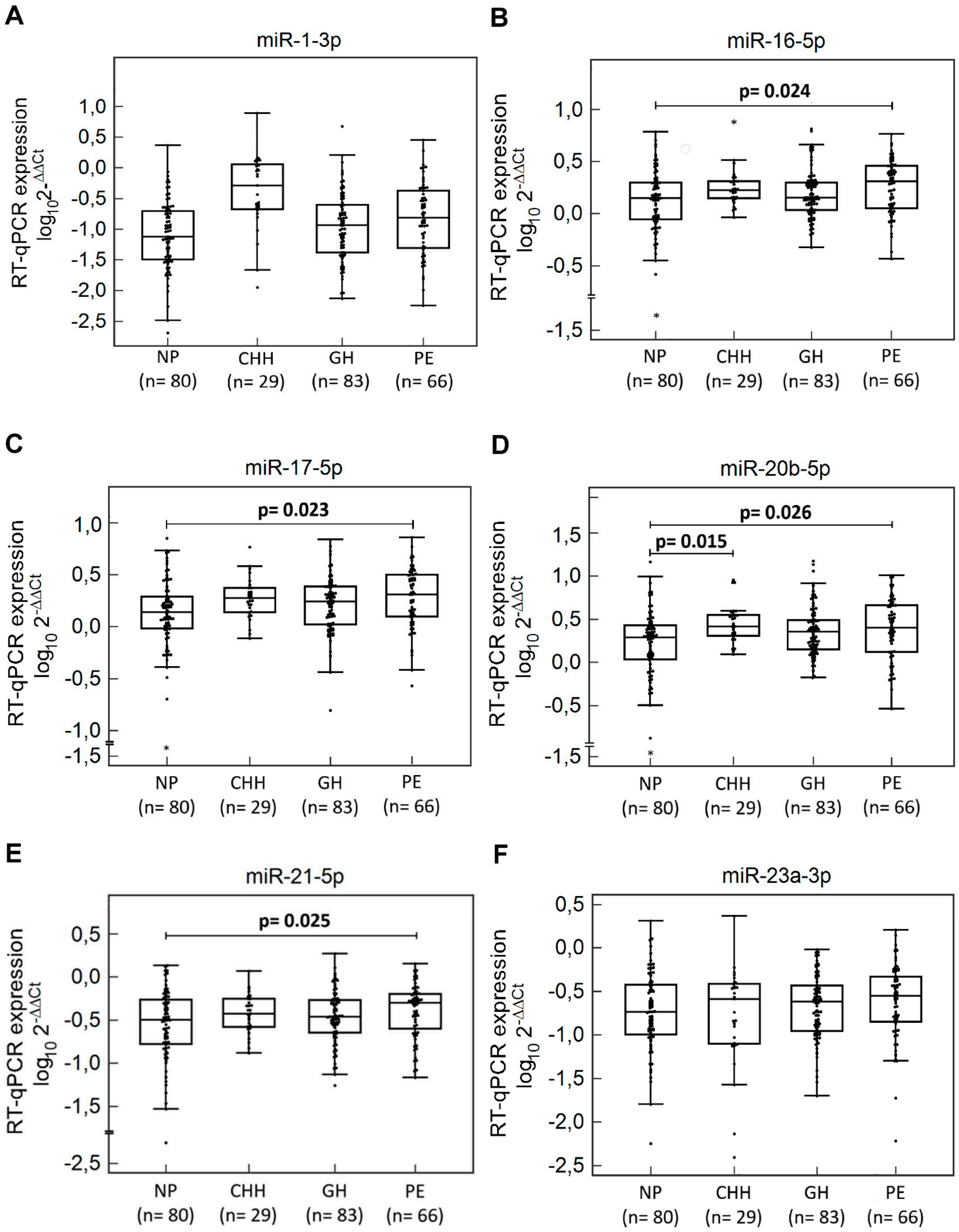

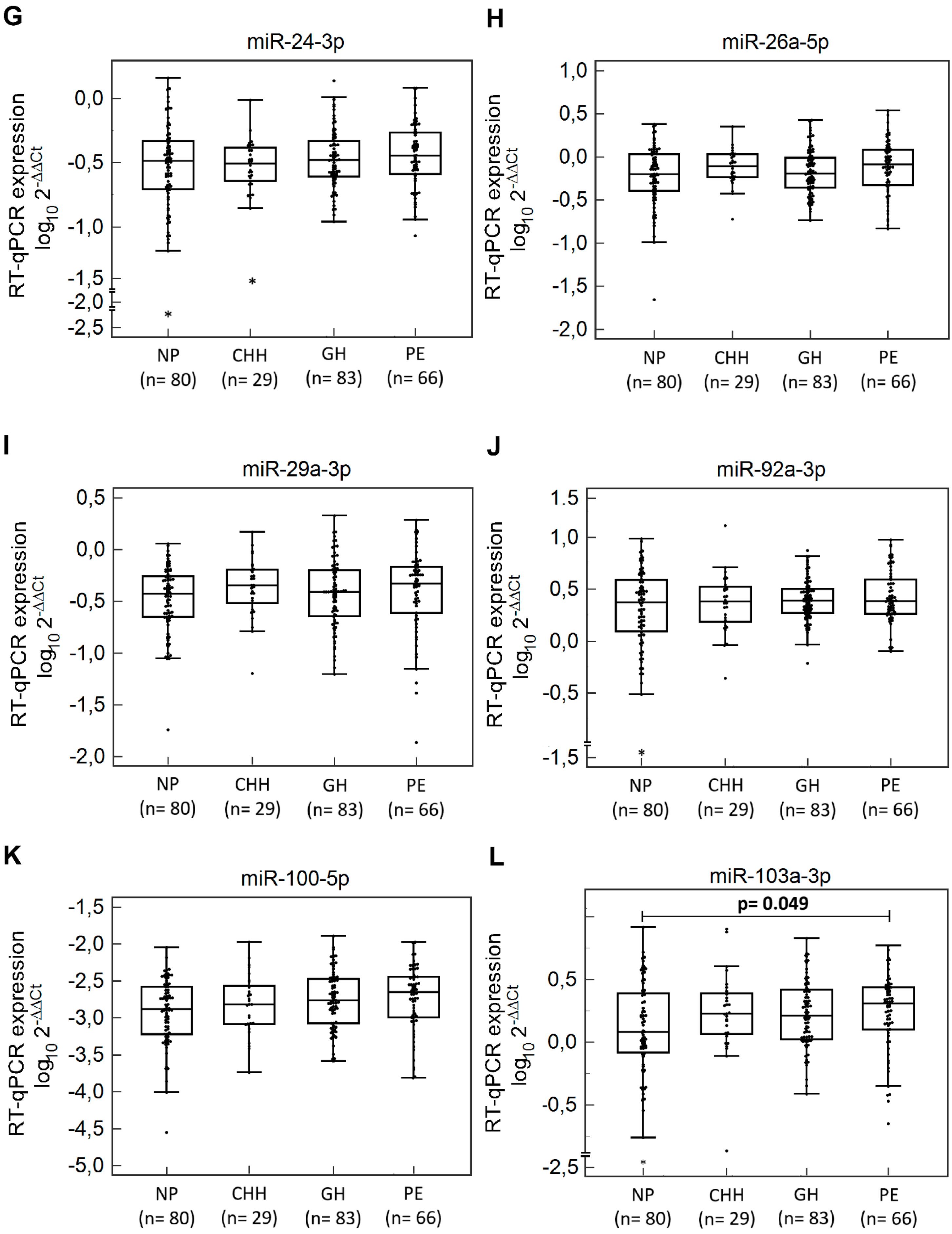
G
miR-24-3p
RT-qPCR expression
log10 2-ΔΔCt
0,0
-0,5
-1,0
-1,5
-2,0
-2,5
NP (n= 80)
CHH (n= 29)
GH (n= 83)
PE (n= 66)
H
miR-26a-5p
RT-qPCR expression
log10 2-ΔΔCt
1,0
0,5
0,0
-0,5
-1,0
-1,5
-2,0
NP (n= 80)
CHH (n= 29)
GH (n= 83)
PE (n= 66)
I
miR-29a-3p
RT-qPCR expression
log10 2-ΔΔCt
0,5
0,0
-0,5
-1,0
-1,5
-2,0
NP (n= 80)
CHH (n= 29)
GH (n= 83)
PE (n= 66)
J
miR-92a-3p
RT-qPCR expression
log10 2-ΔΔCt
1.5
1,0
0,5
0,0
-0,5
-1,5
NP (n= 80)
CHH (n= 29)
GH (n= 83)
PE (n= 66)
K
miR-100-5p
RT-qPCR expression
log10 2-ΔΔCt
-1,5
-2,0
-2,5
-3,0
-3,5
-4,0
-4,5
-5,0
NP (n= 80)
CHH (n= 29)
GH (n= 83)
PE (n= 66)
L
miR-103a-3p
p= 0.049
RT-qPCR expression
log10 2-ΔΔCt
0,5
0,0
-0,5
-2,5
NP (n= 80)
CHH (n= 29)
GH (n= 83)
PE (n= 66)

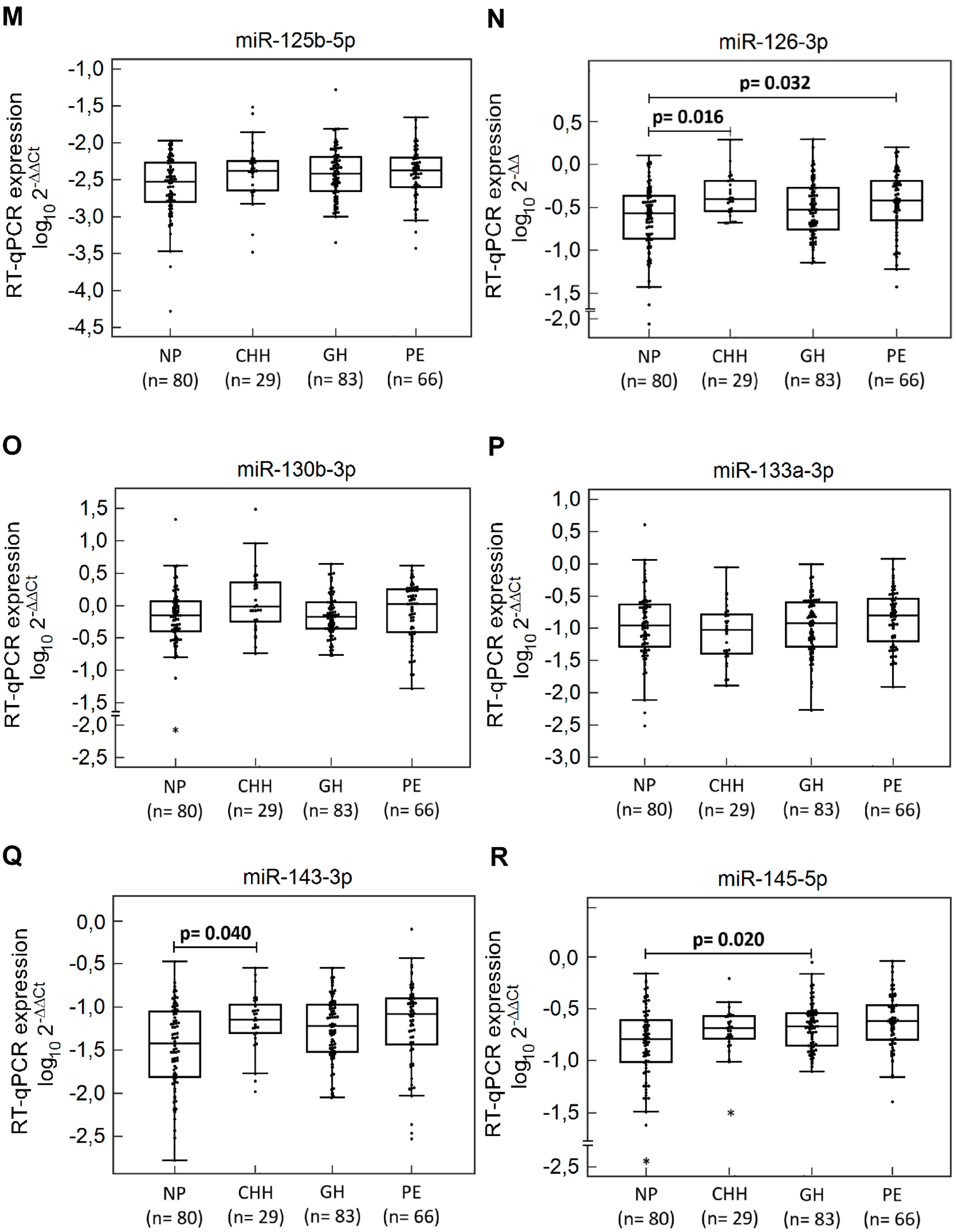
M
miR-125b-5p
RT-qPCR expression
log10 2-ΔΔCt
-1,0
-1,5
-2,0
-2,5
-3,0
-3,5
-4,0
-4,5
NP (n= 80)
CHH (n= 29)
GH (n= 83)
PE (n= 66)
N
miR-126-3p
p= 0.032
p= 0.016
RT-qPCR expression
log10 2-ΔΔ
0,5
0,0
-0,5
-1,0
-1,5
-2,0
NP (n= 80)
CHH (n= 29)
GH (n= 83)
PE (n= 66)
O
miR-130b-3p
RT-qPCR expression
log10 2-ΔΔCt
1,5
1,0
0,5
0,0
-0,5
-1,0
-1,5
-2,0
-2,5
NP (n= 80)
CHH (n= 29)
GH (n= 83)
PE (n= 66)
P
miR-133a-3p
RT-qPCR expression
log10 2-ΔΔCt
1,0
0,5
0,0
-0,5
-1,0
-1,5
-2,0
-2,5
-3,0
NP (n= 80)
CHH (n= 29)
GH (n= 83)
PE (n= 66)
Q
miR-143-3p
p= 0.040
RT-qPCR expression
log10 2-ΔΔCt
0,0
-0,5
-1,0
-1,5
-2,0
-2,5
NP (n= 80)
CHH (n= 29)
GH (n= 83)
PE (n= 66)
R
miR-145-5p
p= 0.020
RT-qPCR expression
log10 2-ΔΔCt
0,0
-0,5
-1,0
-1,5
-2,5
NP (n= 80)
CHH (n= 29)
GH (n= 83)
PE (n= 66)

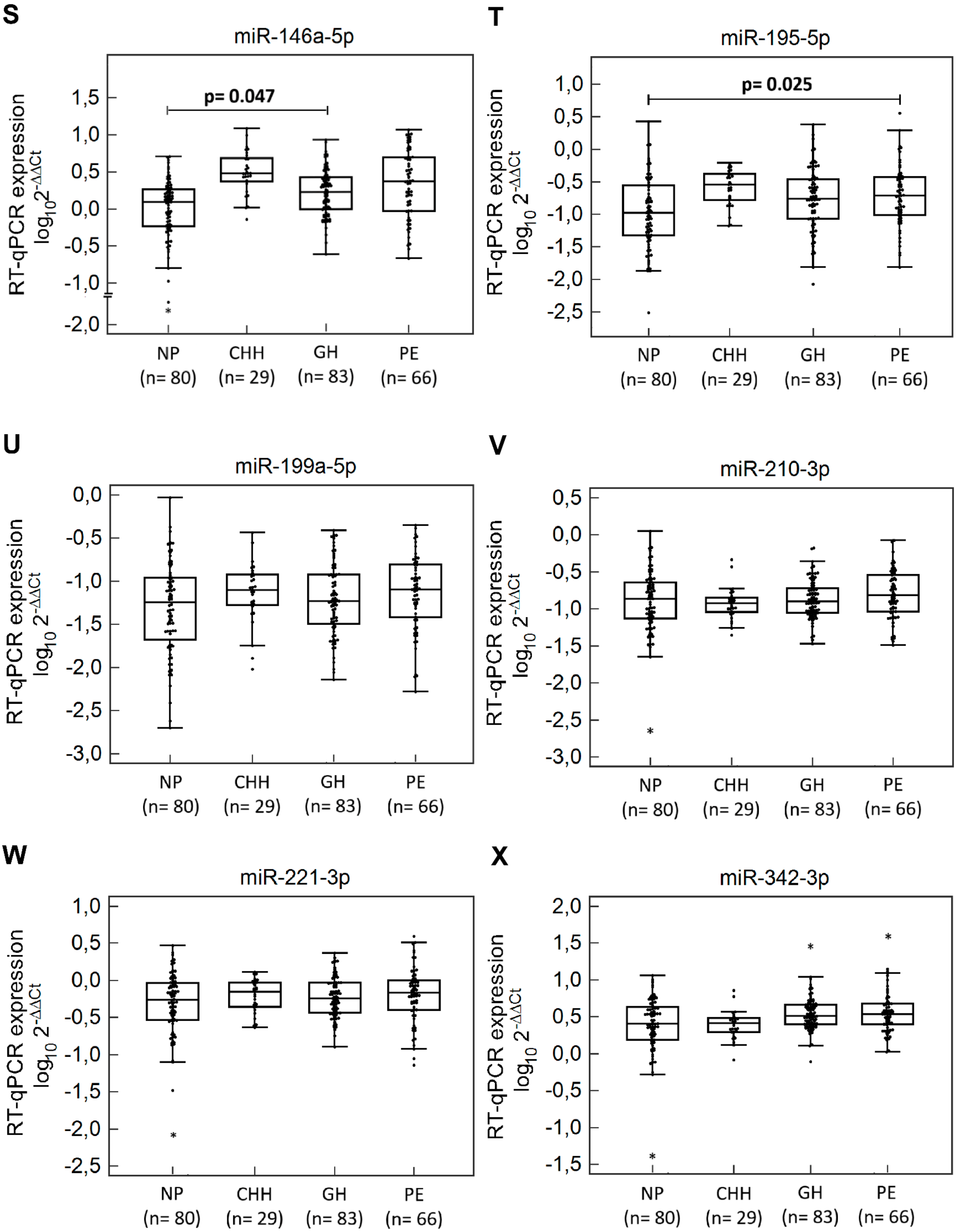
S
miR-146a-5p
p= 0.047
RT-qPCR expression
$\log_{10} 2^{-\Delta\Delta Ct}$
1,5
1,0
0,5
0,0
-0,5
-1,0
-2,0
NP (n= 80)
CHH (n= 29)
GH (n= 83)
PE (n= 66)
T
miR-195-5p
p= 0.025
1,0
0,5
0,0
-0,5
-1,0
-1,5
-2,0
-2,5
U
miR-199a-5p
0,0
-0,5
-1,0
-1,5
-2,0
-2,5
-3,0
V
miR-210-3p
0,5
0,0
-0,5
-1,0
-1,5
-2,0
-2,5
-3,0
W
miR-221-3p
1,0
0,5
0,0
-0,5
-1,0
-1,5
-2,0
-2,5
X
miR-342-3p
2,0
1,5
1,0
0,5
0,0
-0,5
-1,0
-1,5

Y

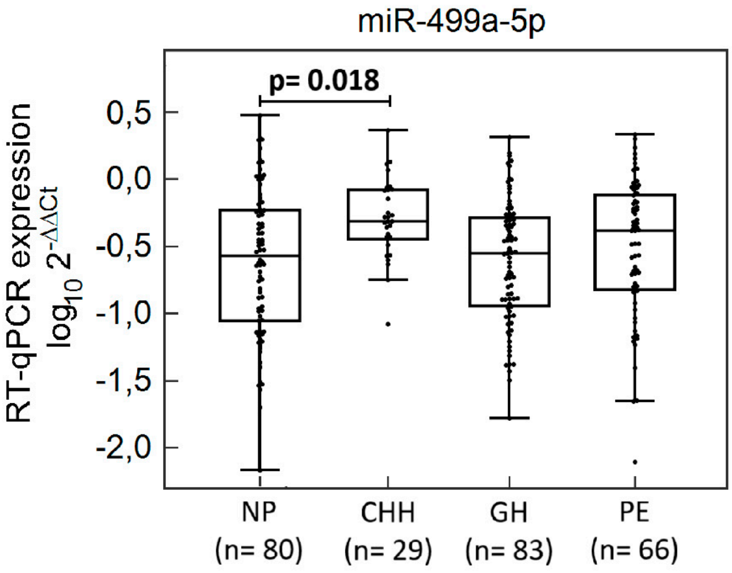

Supplementary Figure S2. Gene expression of cardiovascular disease associated microRNAs in peripheral blood leukocytes in early stages of gestation – comparison between NP, CHH, GH and PE – statistical non-significant data after Benjamini-Hochberg correction.; NP, normal pregnancies; CHH, chronic hypertension; GH, gestational hypertension; PE, preeclampsia.
