# Supplementary material for: Cardiovascular Disease-Associated MicroRNA Dysregulation during the First Trimester of Gestation in Women with Chronic Hypertension and Normotensive Women Subsequently Developing Gestational Hypertension or Preeclampsia with or without Fetal Growth Restriction
Source: Biomedicines. 2022 Jan 25;10(2):256. doi: 10.3390/biomedicines10020256 (PMC8869238; doi:10.3390/biomedicines10020256)
Supplement: Supplementary file 1 [file biomedicines-10-00256-s001.zip › biomedicines-1539289-supplementary/Supplementary Table S1.pdf]

Supplementary Table S1. Correlation between microRNA gene expression and MAP.

microRNA gene expression vs MAP		ϱ (Spearman's rank correlation coefficient)	p-value
miR-1-3p & MAP	mmHg	0.101181	0.130244
	MoM	0.109305	0.102748
miR-16-5p & MAP	mmHg	0.127920	0.055367
	MoM	0.110396	0.099342
miR-17-5p & MAP	mmHg	0.100534	0.132732
	MoM	0.056719	0.398213
miR-20a-5p & MAP	mmHg	0.151779	0.022774
	MoM	0.101216	0.130974
miR-20b-5p & MAP	mmHg	0.083715	0.210960
	MoM	0.045465	0.498402
miR-21-5p & MAP	mmHg	0.115902	0.082794
	MoM	0.082704	0.217580
miR-23a-3p & MAP	mmHg	0.106067	0.112599
	MoM	0.085340	0.203222
miR-24-3p & MAP	mmHg	0.045295	0.499044
	MoM	-0.006524	0.922644
miR-26a-5p & MAP	mmHg	0.085429	0.201734
	MoM	0.052511	0.434182
miR-29a-3p & MAP	mmHg	0.093901	0.160386
	MoM	0.040103	0.550452
miR-92a-3p & MAP	mmHg	0.067669	0.312234
	MoM	0.041251	0.539088
miR-100-5p & MAP	mmHg	0.098287	0.141656
	MoM	0.063888	0.341195
miR-103a-3p & MAP	mmHg	0.132747	0.046710
	MoM	0.094425	0.158992
miR-125b-5p & MAP	mmHg	0.152053	0.022526
	MoM	0.103105	0.123902
miR-126-3p & MAP	mmHg	0.086743	0.194855
	MoM	0.028533	0.671031
miR-130b-3p & MAP	mmHg	0.062253	0.352631
	MoM	0.006059	0.928151
miR-133a-3p & MAP	mmHg	-0.006550	0.922164
	MoM	-0.032215	0.631532
miR-143-3p & MAP	mmHg	0.155871	0.019317
	MoM	0.106393	0.112298
miR-145-5p & MAP	mmHg	0.154970	0.020036
	MoM	0.081479	0.224492
miR-146a-5p & MAP	mmHg	0.196238	0.003117
	MoM	0.121563	0.069379
miR-155-5p & MAP	mmHg	0.206471	0.001849
	MoM	0.159903	0.016609
miR-181a-5p & MAP	mmHg	0.172207	0.009651
	MoM	0.121533	0.069446
	mmHg	0.116547	0.081085

miR-195-5p & MAP	MoM	0.074968	0.263861
miR-199a-5p & MAP	mmHg	0.066260	0.322444
	MoM	0.032103	0.632715
miR-210-3p & MAP	mmHg	0.052606	0.432310
	MoM	0.026058	0.698100
miR-221-3p & MAP	mmHg	0.052716	0.431355
	MoM	-0.015395	0.818769
miR-342-3p & MAP	mmHg	0.120162	0.072032
	MoM	0.036225	0.589672
miR-499a-5p & MAP	mmHg	0.030184	0.652468
	MoM	-0.004077	0.951619
miR-574-3p & MAP	mmHg	0.137673	0.039072
	MoM	0.066085	0.324811

MAP, mean arterial pressure; mmHg, millimetre of mercury; MoM, multiple of median.
