# Supplementary material for: Cardiovascular Disease-Associated MicroRNA Dysregulation during the First Trimester of Gestation in Women with Chronic Hypertension and Normotensive Women Subsequently Developing Gestational Hypertension or Preeclampsia with or without Fetal Growth Restriction
Source: Biomedicines. 2022 Jan 25;10(2):256. doi: 10.3390/biomedicines10020256 (PMC8869238; doi:10.3390/biomedicines10020256)
Supplement: Supplementary file 1 [file biomedicines-10-00256-s001.zip › biomedicines-1539289-supplementary/Supplementary Table S2.pdf]

Supplementary Table S2. Correlation between microRNA gene expression and serum PAPP-A levels.

microRNA gene expression vs serum PAPP-A levels		ϱ (Spearman's rank correlation coefficient)	p-value
miR-1-3p & PAPP-A	IU/l	0.090534	0.148619
	MoM	0.029266	0.642493
miR-16-5p & PAPP-A	IU/l	-0.043601	0.487352
	MoM	-0.129991	0.038425
miR-17-5p & PAPP-A	IU/l	-0.058893	0.347992
	MoM	-0.093929	0.135461
miR-20a-5p & PAPP-A	IU/l	-0.030766	0.624158
	MoM	-0.111785	0.075345
miR-20b-5p & PAPP-A	IU/l	0.019215	0.759637
	MoM	-0.052504	0.404715
miR-21-5p & PAPP-A	IU/l	-0.053628	0.392845
	MoM	-0.098825	0.116161
miR-23a-3p & PAPP-A	IU/l	-0.038479	0.539954
	MoM	-0.088769	0.158379
miR-24-3p & PAPP-A	IU/l	-0.072049	0.250709
	MoM	-0.097069	0.122822
miR-26a-5p & PAPP-A	IU/l	-0.007499	0.904957
	MoM	-0.076819	0.222446
miR-29a-3p & PAPP-A	IU/l	-0.006683	0.915263
	MoM	-0.068365	0.277724
miR-92a-3p & PAPP-A	IU/l	-0.125936	0.044100
	MoM	-0.073608	0.242436
miR-100-5p & PAPP-A	IU/l	0.048187	0.442687
	MoM	-0.055604	0.377510
miR-103a-3p & PAPP-A	IU/l	-0.095912	0.125863
	MoM	-0.083404	0.185173
miR-125b-5p & PAPP-A	IU/l	0.013652	0.827920
	MoM	-0.050310	0.424659
miR-126-3p & PAPP-A	IU/l	-0.068324	0.276105
	MoM	-0.103916	0.098442
miR-130b-3p & PAPP-A	IU/l	-0.007467	0.905358
	MoM	-0.028999	0.645522
miR-133a-3p & PAPP-A	IU/l	0.023851	0.704097
	MoM	0.002056	0.973990
miR-143-3p & PAPP-A	IU/l	-0.009320	0.882033
	MoM	-0.090433	0.150687
miR-145-5p & PAPP-A	IU/l	-0.081073	0.196028
	MoM	-0.079603	0.206079
miR-146a-5p & PAPP-A	IU/l	-0.066113	0.291979
	MoM	-0.126657	0.043722
miR-155-5p & PAPP-A	IU/l	-0.074334	0.235956
	MoM	-0.142412	0.023203
miR-181a-5p & PAPP-A	IU/l	-0.011164	0.858912
	MoM	-0.091416	0.146283
miR-195-5p & PAPP-A	IU/l	-0.001733	0.977986
	MoM	-0.047339	0.452559

miR-199a-5p & PAPP-A	IU/l	0.021752	0.729069
	MoM	-0.016877	0.788961
miR-210-3p & PAPP-A	IU/l	-0.091753	0.143206
	MoM	-0.124836	0.046864
miR-221-3p & PAPP-A	IU/l	-0.077747	0.215072
	MoM	-0.135854	0.030424
miR-342-3p & PAPP-A	IU/l	-0.052063	0.406825
	MoM	-0.051201	0.416492
miR-499a-5p & PAPP-A	IU/l	0.028203	0.653342
	MoM	-0.056869	0.366741
miR-574-3p & PAPP-A	IU/l	-0.001684	0.978604
	MoM	-0.039347	0.532474

PAPP-A, pregnancy-associated plasma protein-A; IU/l, international units per litre; MoM, multiple of median.
